# Breast Cancer Consensus Subtypes: A system for subtyping breast cancer tumors based on gene expression

**DOI:** 10.1038/s41523-021-00345-2

**Published:** 2021-10-12

**Authors:** Christina Horr, Steven A. Buechler

**Affiliations:** 1 Department of Applied and Computational Mathematics and Statistics, University of Notre Dame, Notre Dame, IN USA; 2 Harper Cancer Research Institute, University of Notre Dame, Notre Dame, IN USA

**Keywords:** Breast cancer, Transcriptomics, Diagnostic markers, Cancer genomics

## Abstract

Breast cancer is heterogeneous in prognoses and drug responses. To organize breast cancers by gene expression independent of statistical methodology, we identified the Breast Cancer Consensus Subtypes (BCCS) as the consensus groupings of six different subtyping methods. Our classification software identified seven BCCS subtypes in a study cohort of publicly available data (*n* = 5950) including METABRIC, TCGA-BRCA, and data assayed by Affymetrix arrays. All samples were fresh-frozen from primary tumors. The estrogen receptor-positive (ER+) BCCS subtypes were: PCS1 (18%) good prognosis, stromal infiltration; PCS2 (15%) poor prognosis, highly proliferative; PCS3 (13%) poor prognosis, highly proliferative, activated IFN-gamma signaling, cytotoxic lymphocyte infiltration, high tumor mutation burden; PCS4 (18%) good prognosis, hormone response genes highly expressed. The ER− BCCS subtypes were: NCS1 (11%) basal; NCS2 (10%) elevated androgen response; NCS3 (5%) cytotoxic lymphocyte infiltration; unclassified tumors (9%). HER2+ tumors were heterogeneous with respect to BCCS.

## Introduction

Gene expression-based signatures and subtyping systems are widely recognized as valuable methods for disease stratification in breast cancer. Several signatures^1–4^ are in clinical use for prognosis and treatment selection for estrogen receptor-positive (ER+) breast cancer. The intrinsic subtypes^5^, also known as the PAM50 subtypes, are commonly used to describe molecular features of tumors in addition to hormone receptor and HER2 status. Several groups have developed subtype systems specific to the clinically important triple-negative breast cancers (TNBC); i.e., tumors that are estrogen receptor-negative (ER−), progesterone receptor-negative (PR−), and HER2- (TNBC)^6–8^. Lehmann et al. identified six subtypes, Burstein et al. identified four subtypes, and Jézéquel et al. presented three subtypes. The clinical translation and utility of these systems could be hampered by these discordant results, likely due to diverse algorithms and patient cohorts.

The colorectal cancer community also faced the problem of multiple validated gene expression-based subtyping systems. In the absence of a gold-standard method, the Colorectal Cancer Subtyping Consortium (CRCSC), published the consensus molecular subtypes (CMS) of colorectal cancer^9^. The CRCSC used network cluster analysis to form subtypes that drew on information from six independent subtype systems. In a sense, two samples were classified into the same consensus molecular subtype if the six systems largely agreed that they should be classified together. This methodology had the effect of minimizing bias due to the clustering method or training cohort.

Current subtyping results for breast cancer are also limited in their abilities to inform how non-TNBC tumors respond to treatments. For example, results have shown differing sensitivity to therapeutics depending on the concentration of tumor-infiltrating lymphocytes (TILs) in HER2+ tumors^10–13^, evidence of the heterogeneity of HER2+ tumors. Moreover, current subtyping methods for ER+ breast cancers have not identified differential sensitivity to CDK4/6 inhibitors.

In this study, we developed a gene expression-based subtype system for breast cancer by adapting the methods leading to the colorectal cancer CMS subtypes. We chose to subtype breast cancers overall, rather than restricting attention to TNBC tumors, to avoid unnecessarily restricting the scope of the work. Following the derivation of the Breast Cancer Consensus Subtypes (BCCS) and a computer application to generate the subtypes, we identified BCCS in cohorts based on three different gene expression platforms and analyzed the cohort-independent biological and clinical features of the subtypes.

## Results

### Study cohorts

**Fig. 1:**
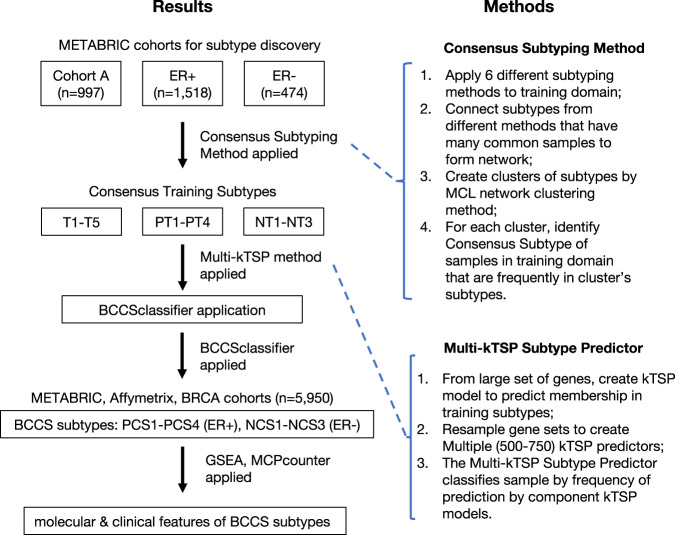
Project workflow. The analysis workflow for the project is diagramed, describing both the results and the novel methods applied.

The BCCS subtypes were studied in the cohorts METABRIC (*n* = 1992), Affymetrix (*n* = 2923), and BRCA (*n* = 1035), with gene expression based on Illumina bead array, Affymetrix microarray, and RNA-sequencing, respectively (see the “Methods” section, Supplementary Table 1). All samples were from primary tumors and fresh-frozen tissue. METABRIC was partitioned into METABRIC-A (*n* = 997), and METABRIC-B (*n* = 995) using the discovery-validation partition of Curtis et al.^14^. BCCS subtypes were trained in METABRIC-A and their features were explored in all three cohorts (Fig. 1).

### Discovery of consensus training subtypes

**Fig. 2:**
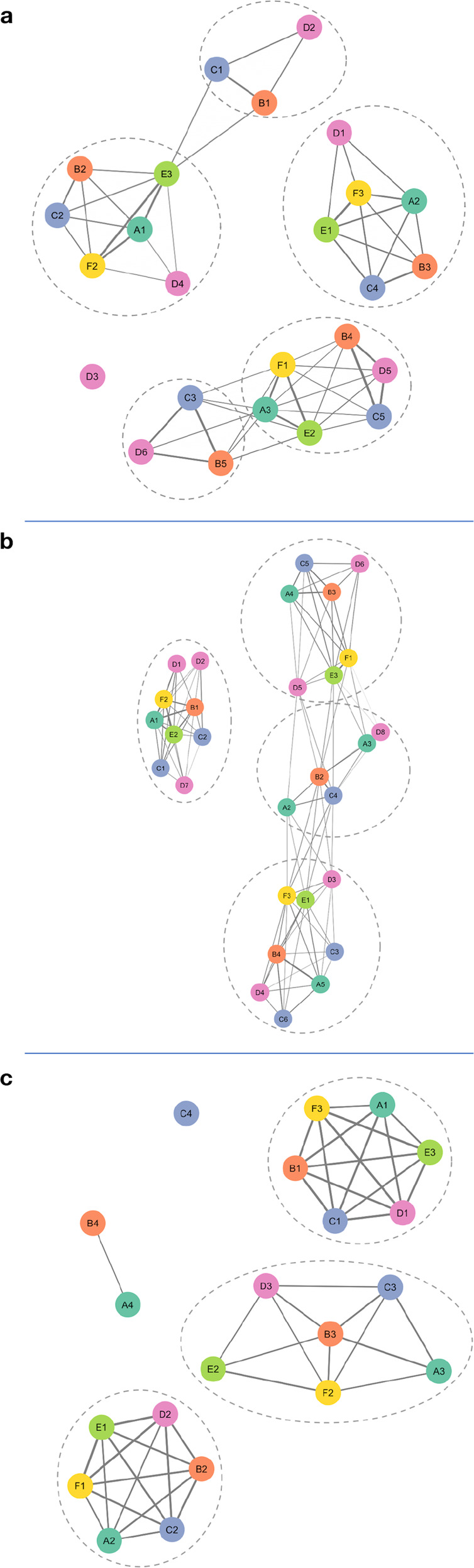
Networks of subtypes produced by six different methods. The networks are displayed for **a** METABRIC-A, **b** METABRIC ER−, **c** METABRIC ER+. The nodes are colored and labeled according to the particular subtyping method. An edge between nodes indicates that the corresponding subtypes had a significant number of samples in common. Dashed circles indicate the nodes (subtypes) that were clustered together by the MCL network clustering method. **a** Application of methods A–F resulted in subtype systems with 3, 5, 5, 6, 3, 3 subtypes, respectively. The three subtypes identified by Method A were labeled A1–A3, and similarly for other methods. A network was formed having nodes these 25 subtypes. Markov clustering of this network resulted in five clusters of subtypes (identified with dashed ovals), plus the singleton D3 (*n* = 31). For each cluster of subtypes, a subset of METABRIC-A (labeled T1–T5) that formed the core samples of the cluster was identified, with remaining samples in T0 (*n* = 92, 9.2%). **b** Methods A–F applied to this domain resulted in 4, 4, 4, 3, 3, 3 subtypes, respectively. Markov cluster analysis of the network of 21 subtypes identified 3 clusters of subtypes, with three unclustered subtypes. Core samples assigned to each cluster formed NT1–3 with unclassified samples comprising NT0. **c** In METABRIC ER+ samples, Methods A–F resulted in 5, 4, 6, 8, 3, 3 clusters, respectively. Markov cluster analysis applied to the network of 29 clusters resulted in four clusters that grouped 9, 5, 8, and 7 nodes, respectively. Core sample assignment to these clusters created subtypes PT1–4 and unclassified samples as PT0.

The consensus subtyping method (see the “Methods” section) was first applied to the METABRIC-A cohort, which includes both ER+ and ER− samples. Six gene filtering and subtyping methods (A–F) were selected to mirror those used in consensus molecular subtyping of colorectal cancers^9^ (see the “Methods” section). Application of the consensus subtyping method (Fig. 2a) resulted in subsets T1–T5 of METABRIC-A, called the BC Consensus Training Subtypes, with unclassified samples labeled T0. The subtypes T1, T2 consisted predominately of ER− samples, while T3–T5 consisted predominately of ER+ samples (Supplementary Table 2). Because these clusters largely separated ER+ and ER− samples (accuracy = 0.96), a fortiori, we explored the potential to identify additional heterogeneity by first separating samples by ER status, and then repeating the consensus subtyping method separately for ER− samples and ER+ samples.

Consensus subtyping applied to the METABRIC ER− samples (*n* = 474, Fig. 2b) resulted in subtypes NT1 (*n* = 193), NT2 (*n* = 183), NT3 (*n* = 73), called the ER− Consensus Training Subtypes, with unclassified samples NT0 (*n* = 25). (All ER− samples in METABRIC were used to provide a sufficiently large training set.) Consensus subtyping applied to the METABRIC ER+ samples (*n* = 1518, Fig. 2c), resulted in clusters PT1 (*n* = 440), PT2 (*n* = 316), PT3 (*n* = 354), and PT4 (*n* = 351), called the ER+ Consensus Training Subtypes, with unclassified samples PT0 (*n* = 57).

### Derivation of BCCSclassifier, a computer application to generate BCCS in whole-transcriptome datasets

It would be impractical to apply the consensus subtyping process used above in an arbitrary cohort, and the method cannot be applied to a single new sample. To resolve these problems, computer applications were trained to the BC, ER+ and ER− Consensus Training Subtypes, using the Multi-kTSP generalization of kTSP (see the “Methods” section). Predictors of T1–T5 were defined on a discovery set of 75% of the METABRIC-A cohort and evaluated in the complementary test set (25% of samples) as predictors of T1–T5. Accuracy of these predictors reached a plateau of 92% in the test set using a predictor based on 500 kTSP models. This application was called BCCSclassifier(ER+/−). The sets of samples predicted to be in T1–T5 by BCCSclassifier(ER+/−) in any cohort were called Breast Consensus Subtype 1–5 (BCS1–BCS5), respectively.

This process was repeated to define a predictor of PT1–PT4 in the METABRIC ER+ samples. Using 750 predictors achieved an accuracy of 83% in a test subset of 25% of the samples. The subtypes this application [BCCSclassifer(ER+)] predicted to be in PT1–PT4 in a sample set were called estrogen-receptor *P*ositive *C*onsensus *S*ubtype 1–4 (PCS1–PCS4), respectively. We similarly derived the application BCCSclassifer(ER−) which computed *N*egative *C*onsensus *S*ubtype 1–3 (NCS1–NCS3) by training to NT1–NT3. Samples unclassified by these applications were labeled “tie”.

The BCS, PCS, and NCS subtype assignments for all samples in METABRIC, Affymetrix, and BRCA cohorts are delineated in Supplementary Data 1 with distributions in Supplementary Table 3. Of the pairs clustered together by BCS, 92.4% had the same ER status; i.e., BCS separated samples by ER status, a fortiori. Because subtyping ER+, respectively ER−, tumors into PCS1–4, respectively NCS1–3, was more refined than BCS1–5, the further analysis focused on PCS1–4, and NCS1–3. Collectively, we called PCS1–4 and NCS1–3 the BCCS.

### Test of the robustness of subtypes to alternative method of derivation

The six methods employed in the derivation of the BCCS subtypes produced subtypes that potentially varied in the number of subtypes and may have disagreed in classifications (Fig. 2). The consensus subtyping method aims to cluster a pair of samples only when multiple systems using different methods clustered them together, and in this sense generates clusters that are less dependent on subtyping method than a single system. To test the degree of robustness of the BCCS subtypes, we repeated the derivation for the ER+ subtypes, the most complex case, using an alternative set of subtyping systems (see the “Methods” section). The alternative six methods produced systems with 6, 3, 4, 5, 4, and 6 subtypes. However, application of the consensus subtyping method resulted in four subtypes that reproduced PCS1–4 in METABRIC ER+ with accuracy = 0.92 and kappa = 0.90, providing evidence that the BCCS classifications are robust to changes in the clustering method.

### Distributions of clinico-pathological traits and PAM50 subtypes in the BCCS subtypes

As an initial exploration of the features of the BCCS subtypes, we computed the proportions of samples in each subtype having selected clinico-pathological traits for METABRIC and BRCA (Supplementary Table 4). (Clinical data was unavailable for many patients in Affymetrix cohort.) Among the ER+ samples in METABRIC, the proportion of grade 3 tumors in PCS2 and PCS3 was 0.51 and 0.69, respectively, which was significantly higher (*p* = 3 × 10^−48^) than the proportions in PCS1 (0.23) and PCS4 (0.20), combined. Similarly, PR− ER+ tumors were more likely to be in PCS2 and PCS3 than PCS1 and PCS4 (*p* < 0.0001 for all comparisons). The BCCS subtypes did not exhibit notable differences in proportions of LN+ tumors, tumors at least 2 cm in size, or patients over 50. The relationship between BCCS and HER2 status is discussed in depth below.

The BCCS subtypes for ER+ samples varied significantly in distant metastasis-free survival estimates in METABRIC (*p* = 6 × 10^−11^, Supplementary Fig. 1A) and Affymetrix cohort (*p* = 5 × 10^−16^, Supplementary Fig. 2A). In both cohorts, each of PCS2 and PCS3 had poorer prognosis than PCS1 and PCS4 (*p* < 0.001 for all Cox proportional hazards models), and prognosis was not significantly different between PCS2 and PCS3 (*p* = 0.06 in METABRIC, *p* = 0.2 in Affymetrix). Prognosis for the BCCS ER− subtypes varied slightly for METABRIC (*p* = 0.02, Supplementary Fig. 1B), and not significantly for Affymetrix (*p* = 0.72, Supplementary Fig. 2B).

Lobular carcinomas were disproportionately found in PCS1 (124/238 in METABRIC, 130/197 in BRCA; see Supplementary Table 4 for prevalence in all BCCS) and mucinous carcinomas were disproportionately found in PCS4 (23/46 in METABRIC, 13/16 in BRCA).

The interaction between BCCS and PAM50 subtypes was tabulated for METABRIC and BRCA (Table 1). Of note, the majority of Luminal B samples were in PCS2 or PCS3, the majority of Luminal A were in PCS1 or PCS2, and most Normal were in PCS1. NCS2 samples were largely Her2 or Normal, while NCS3 samples were from Basal or Her2. These results provided evidence that the BCCS subtypes could not be derived from the PAM50 subtypes and clinico-pathological covariates.

**Table 1:** Distribution of samples classified by both BCCS and PAM50 subtypes for METABRIC and BRCA cohorts.

METABRIC (*n* = 1992)						
	Basal	Her2	LumA	LumB	Normal	NC^a^
NCS1	190	0	0	0	3	0
NCS2	38	126	4	0	20	0
NCS3	61	16	0	0	15	0
PCS1	8	5	296	31	121	1
PCS2	1	45	114	185	11	2
PCS3	30	42	47	182	17	1
PCS4	0	4	233	89	11	1
tie	3	2	27	5	4	1
**BRCA (*n*** **=** **1035)**						
NCS1	139	0	0	0	1	0
NCS2	11	46	11	3	9	0
NCS3	11	1	0	0	4	0
PCS1	2	4	271	9	19	1
PCS2	0	3	22	52	2	8
PCS3	16	18	14	66	1	0
PCS4	0	1	158	38	0	2
tie	2	3	58	27	1	1

^a^Not classified.

### Features of gene expression in BCCS subtypes by gene set enrichment analysis (GSEA) and analysis of specific genes

**Fig. 3:**
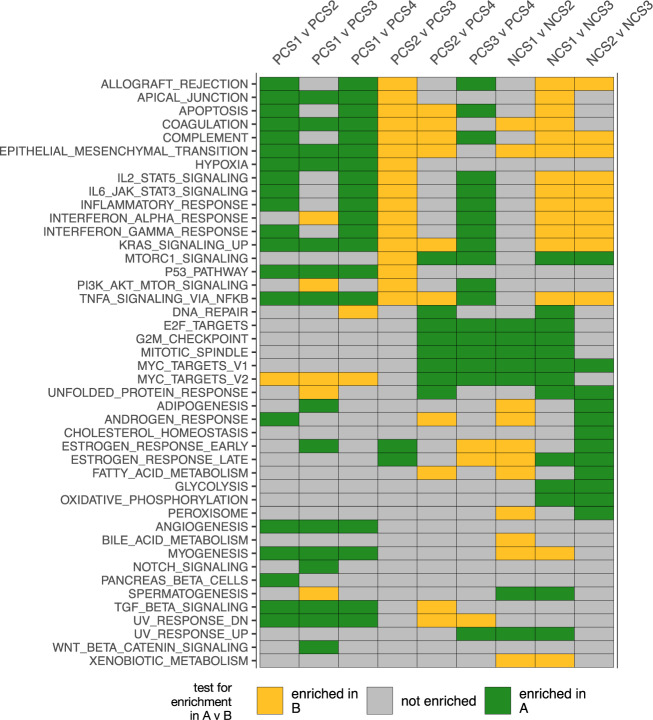
Gene set enrichment by GSEA for pairs of BCCS subtypes. Enrichment for each Hallmark v7 gene set was tested between each pair of PCS1–4 and NCS1–3 and all three study cohorts using GSEA. A gene set was considered enriched between a pair of subtypes if the adjusted *p*-value was < 0.05 in all three cohorts. A GSEA test for enrichment in A versus (v) B reported the normalized enrichment score (NES); NES was positive if expression was enriched in A compared to B, and NES was negative if expression was enriched in B compared to A. A Hallmark gene set is omitted if it was not significantly enriched in any test. A tile is colored green (respectively, goldenrod) for the given pair and gene set, if in all three study cohorts, the adjusted *p*-value was < 0.05 and NES was positive (respectively, negative); a tile is gray if the adjusted *p*-value was ≥ 0.05 in some cohort.

GSEA was used to uncover subtype-specific biological activity. Specifically, enrichment for each Hallmark v7 gene set was tested between each pair of PCS1–4 and NCS1–3 in all three study cohorts (Fig. 3). Multiple gene sets related to cancer development and progression were significantly enriched between subtypes in all three study cohorts. The ER+ subtypes with the poorest prognosis, namely PCS2 and PCS3, were enriched in MYC targets (V2) compared to PCS1 and PCS4, and also E2F targets and G2M checkpoint compared to PCS4. From these results, we conclude that tumors in both PCS2 and PCS3 were, on average, highly proliferative^15^. However, significant differences between PCS2 and PCS3 were also observed. Compared to PCS2, PCS3 was enriched in interferon-gamma (IFN-*γ*) response, IL6-JAK-STAT3 signaling, complement, apoptosis, P53 pathway, and other pathways related to immune response and hypoxia, while PCS2 was enriched in estrogen response compared to PCS3.

The two good prognosis ER+ subtypes, namely PCS1 and PCS4, also varied in gene set enrichment compared to other subtypes. Compared to PCS2-4, PCS1 was enriched in TGF-*β* signaling, angiogenesis, coagulation, and other pathways reflecting stromal cell infiltration. PCS4 was enriched in MYC targets (V2) compared to PCS1.

Turning to the ER− subtypes, NCS1 was enriched in E2F targets, G2M checkpoint and MYC targets compared to NCS2 and NCS3, typical of basal-like tumors; NCS2 was enriched in androgen response and estrogen response compared to NCS1 and NCS3; and NCS3 was enriched in IFN-*γ* response, IL6-JAK-STAT3 signaling, complement, and other immune response gene sets compared to NCS1–2. Since all tumors in these comparisons were ER−, we examined expression levels of ESR1, PGR, AR, and estrogen response genes most significantly varying with respect to BCCS (Supplementary Fig. 3). While expression levels of ESR1 and PGR were comparably low in all of NCS1–3, expression levels of AR, CA12, TFF3, and XBP1 were significantly higher in NCS2 than NCS1 and NCS3 (*p* < 10^−80^ for all tests).

Mean expression levels of hormone response genes also varied across subtypes of ER+ tumors (Supplementary Fig. 3). In particular, mean expression of ESR1 in PCS4 was significantly higher than in each other subtype in all cohorts (*p* < 0.01 for all comparisons). Of note, the mean expression of ESR1 was also significantly lower in each of PCS1 and PCS3 than PCS2 (*p* < 10^−12^ for all comparisons). Also, PCS3 samples in BRCA were more likely to be <90% positive for ER by immunohistochemistry (*p* = 2.1 × 10^−8^), PCS4 samples were more likely to be ≥ 90% positive (*p* = 1.5 × 10^−10^).

**Fig. 4:**
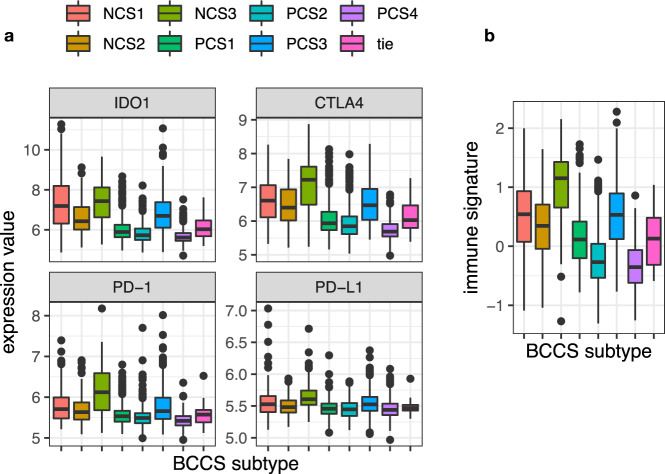
Distribution by BCCS subtypes for immune regulatory genes, and 18-gene immune signature. **a** Distributions are plotted by BCCS subtypes of METABRIC for the immune regulatory genes IDO1, CTLA4, PD-1 (PDCD1) and PD-L1 (CD274). **b** The distributions are plotted by BCCS subtypes of METABRIC of the 18-gene immune signature. Higher values of the score predicted greater sensitivity to pembrolizumab in a previous study. The midline in a boxplot indicates the median, the upper and lower edges indicate the quartiles, and the whisker lines 1.5 times the interquartile range.

The sensitivity of a tumor to immune checkpoint inhibitors (ICI) depends on the levels of checkpoint inhibitor proteins in the tumor, as well as other features of immune activity^16^. In METABRIC and BRCA, expression levels of the checkpoint regulatory genes IDO1, CTLA4, PD-1 (PDCD1), and PD-L1 (CD274) were all significantly higher in NCS3 compared to other samples (*p* < 1.5 × 10^−3^ for all tests), and significantly higher in PCS3 compared to other ER+ samples (*p* < 10^−11^ for all tests, see Fig. 4a for METABRIC). An 18-gene immune signature was previously shown to predict response to pembrolizumab^16^ in multiple tumor types, including TNBC. Values of this immune signature were highest in NCS3 in METABRIC overall (*p* = 1.4 × 10^−31^), and highest in PCS3 compared to other ER+ METABRIC tumors (*p* = 1.5 × 10^−63^, Fig. 4b).

### Relationships between BCCS subtypes and HER2 status

The intrinsic subtype system (PAM50) defined the Her2-enriched subtype to represent all HER2+ tumors. In contrast, HER2+ tumors were found in multiple BCCS subtypes in METABRIC and BRCA (Supplementary Table 5). In METABRIC ER+ the distribution of tumors with HER2 gain was: PCS1 (16%), PCS2 (33%), PCS3 (42%), PCS4 (9%). Previously, the enrichment levels in biological pathways between the BCCS subtypes were analyzed with GSEA. To test the possible dependence of these features on HER2 status we repeated GSEA analysis separately for HER2+ and HER2− tumors in METABRIC for those subtypes with a sufficient number of tumors of each type, specifically, PCS1–3. Results showed (Supplementary Fig. 4) that for most gene sets, the degree of enrichment between subtypes was the same in HER2+ and HER2− tumors. For example, HER2+ tumors in PCS3 were enriched in IFN-*γ* response compared to HER2+ tumors in PCS2, and HER2+ PCS2 tumors were enriched in estrogen response compared to HER2+ PCS3 tumors. The same relationships held for HER2− PCS2 and PCS3 tumors. Thus, the BCCS subtypes articulated heterogeneity in HER2+ tumors and HER2− tumors alike.

While relationships between BCCS subtypes did not significantly vary by HER2 status, features of samples within a subtype did vary by HER2 status. Application of GSEA showed that within each of PCS1, PCS2 and PCS3, HER2+ tumors were enriched for, e.g., MYC targets, and mTOR signaling in comparison to HER2− tumors, providing further evidence that HER2 status and BCCS provide independent information on tumor biology.

A subtype of androgen-receptor-positive, luminal-like tumors (LAR) has previously been identified in TNBC^6–8^, as well as AR+, ER−, HER2+ breast cancers^17^. The subtype NCS2 was enriched in androgen response and included both TNBC and HER2+ samples.

### Degrees of immune and stromal cell infiltration, and tumor cellularity in BCCS subtypes

**Fig. 5:**
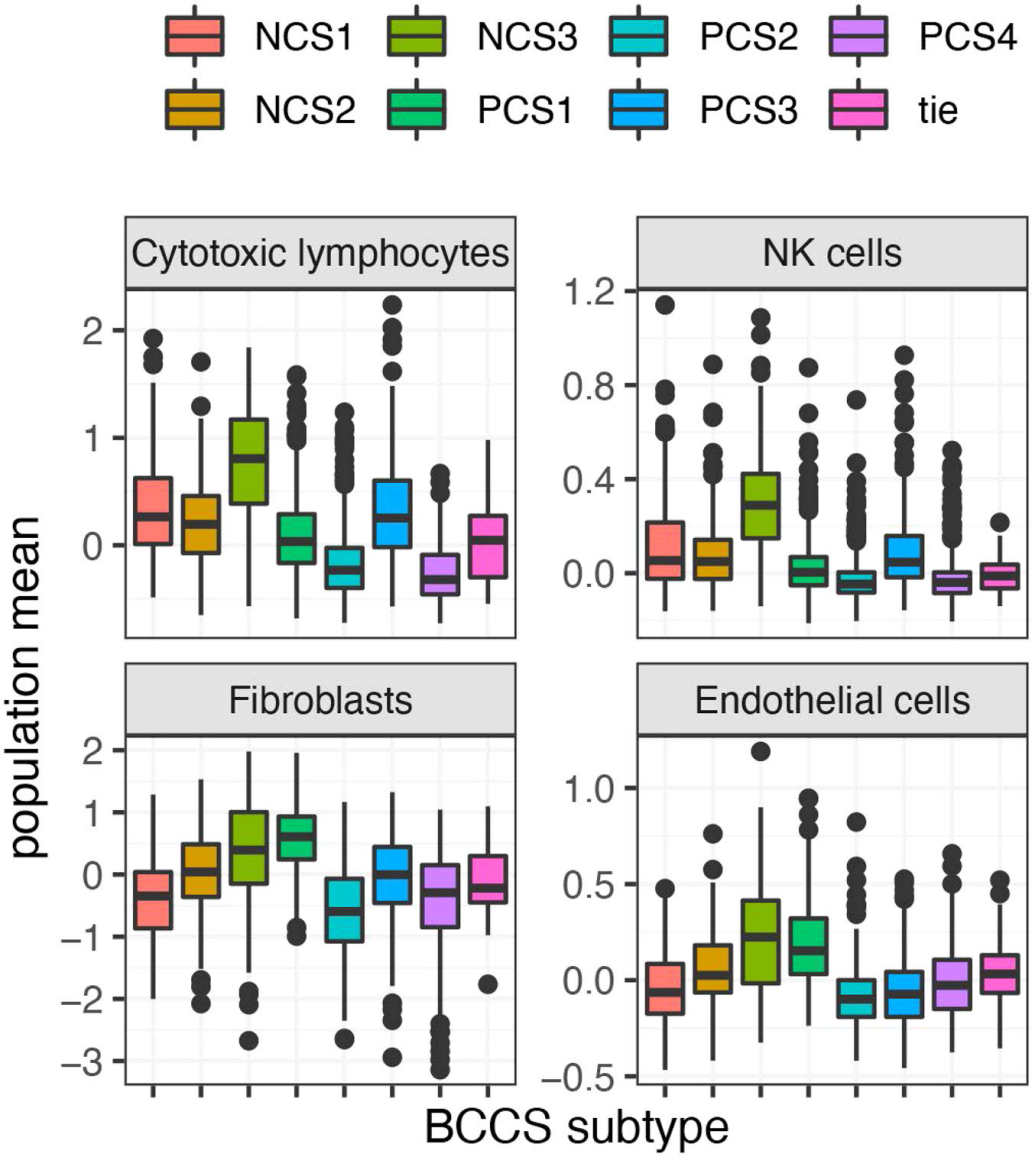
Distribution by BCCS subtypes of measures of infiltration of selected immune and stromal cell populations. This figure plots the distributions by METABRIC BCCS subtypes for the degrees of infiltration by cells of selected populations, specifically, cytotoxic lymphocytes, NK cells, fibroblasts, and endothelial cells. The degree of infiltration by a cell population was assessed with the median-centered MCPcounter population means. The midline in a boxplot indicates the median, the upper and lower edges indicate the quartiles, and the whisker lines are 1.5 times the interquartile range.

TILs in breast cancers have been associated with response to chemotherapy and targeted therapy for HER2+ breast cancer. The distributions of the infiltration levels of immune and stromal cell populations computed by MCPcounter (see the “Methods” section), were assessed with respect to the BCCS subtype (Supplementary Fig. 5). Analysis of BCCS subtypes in METABRIC for selected populations (Fig. 5) showed that NCS3 had significantly greater infiltration of cytotoxic lymphocytes (*p* = 7.4 × 10^−29^) and natural killer cells (*p* = 3.5 × 10^−29^) compared to all other tumors. With respect to ER+ tumors, PCS3 exhibited significantly greater infiltration of cytotoxic lymphocytes (*p* = 6.3 × 10^−44^) and natural killer cells (*p* = 3.3 × 10^−29^) compared to other ER+ tumors. Mean infiltration of fibroblasts was greatest overall in PCS1 (*p* = 6 × 10^−100^), and greatest in NCS3 (*p* = 2.6 × 10^−8^) among ER− tumors. Endothelial cell infiltration was greatest in NCS3 or PCS1 in all three cohorts. Distributions for selected cell populations for Affymetrix and BRCA were similar (Supplementary Fig. 6).

Tumors with low or moderate cellularity in METABRIC were disproportionately found in PCS1 (Supplementary Table 6, *p* = 7.7 × 10^−21^). This relationship was supported by the ASCAT tumor purity distribution in BRCA (Methods, Supplementary Fig. 7, *p* = 2.0 × 10^−39^).

### Relationships between BCCS subtypes and alterations of DNA

**Fig. 6:**
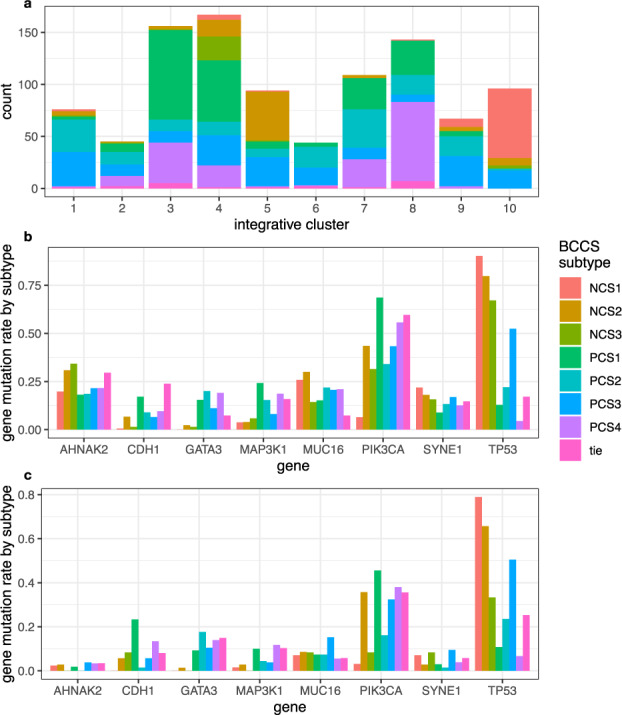
Distribution of copy number changes and somatic mutations by BCCS subtype. The distribution by BCCS subtype of Integrative Cluster of copy number alterations in METABRIC-A is displayed in (**a**). Distribution by BCCS subtype of somatic mutations of AHNAK2, CDH1, GATA3, MAP3K1, MUC16, PIK3CA, SYNE1, TP53 is displayed for **b** METABRIC and **c** BRCA.

Patterns of copy number alterations, mutations of specific genes, tumor mutation burden, and DNA methylation were assessed for correlation with BCCS. The integrative clusters^14^ (IntClust 1–10) grouped samples in METABRIC-A by patterns of copy number changes. The distribution of BCCS subtypes within these clusters showed several significant correlations (Fig. 6a). IntClust 3, characterized by gain in 1q, was enriched in PCS1 (*p* = 5 × 10^−24^); IntClust 8 (1q gain, 16q loss) was enriched in PCS4 (*p* = 1 × 10^−32^); IntClust 10, a high genomic instability group typical of basal tumors (5 loss/8q gain/10p gain/12p gain), was enriched in NCS1 (*p* = 1 × 10^−115^); IntClust 5, associated with ERBB2 amplification, was enriched in NCS2, PCS2 and PCS3 (*p* = 1 × 10^−18^); IntClust 6 (8p12 loss) and IntClust 7 (16q loss, 8q gain) were enriched in PCS2 (*p* = 2 × 10^−11^, *p* = 2 × 10^−6^, respectively). For chromosome arms most altered in integrative clusters, we selected as representative cytobands those from the chromosome arm most significantly altered in METABRIC-A. Copy number changes for representative cytobands in BRCA were consistent with these patterns (Supplementary Table 7).

Mutation status for a selected set of genes (*n* = 173) has been assessed for METABRIC samples (see the “Methods” section). There were eight genes mutated in at least 20% of samples in some BCCS subtypes in METABRIC (Fig. 6b). The mutation frequencies by BCCS subtype were also computed for these genes in BRCA (Fig. 6c). Mutation rates for TP53 were greatest in NCS1 (0.67 in METABRIC, 0.79 in BRCA), and greatest among ER+ tumors in PCS3 (0.52 in METABRIC, 0.50 in BRCA). In the other poor prognosis ER+ subtype, PCS2, TP53 mutation rate was significantly lower (0.22 in METABRIC, *p* = 5 × 10^−16^; 0.24 in BRCA, *p* = 0.0004). PCS1 exhibited the greatest mutation rates for PIK3CA (0.69 in METABRIC, 0.46 in BRCA), CDH1 (0.20 in METABRIC, 0.23 in BRCA), and MAP3K1 (0.24 in METABRIC, 0.1 in BRCA). Of note, mutations in CDH1 have been associated with lobular breast cancer^18^, which is most frequent in PCS1.

The degree of genomic instability, as represented by tumor mutation burden (TMB, see the “Methods” section), varied significantly by BCCS subtypes in BRCA (Supplementary Fig. 8, *p* = 1.5 × 10^−37^). Of note, among ER+ tumors, TMB was significantly elevated (*p* = 1 × 10^−16^) in PCS3, and TMB was not significantly different across NCS1, NCS2, and PCS3 (*p* = 0.18). Given the elevated immune activity in NCS3 the low level of TMB was surprising, however, this could be an aberration due to the small number of NCS3 samples in BRCA (*n* = 16).

**Fig. 7:**
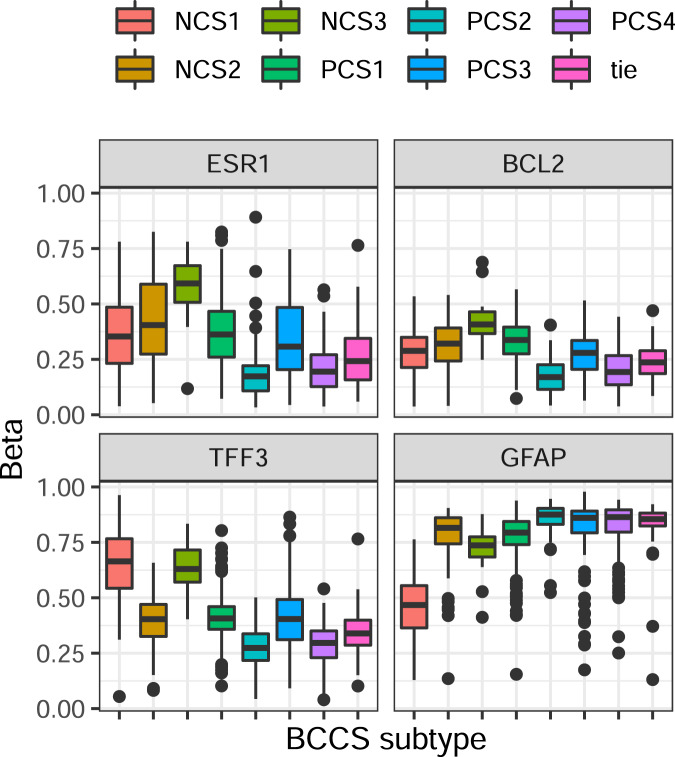
Distribution of methylation levels for selected genes by BCCS subtypes in BRCA. Methylation Beta values are displayed for promoter regions of ESR1 (cg15626350), BCL2 (cg24408313), TFF3 (cg04806409), and GFAP (cg21944455). The midline in a boxplot indicates the median, the upper and lower edges indicate the quartiles, and the whisker lines are 1.5 times the interquartile range.

Methylation in the promoter of ESR1 has been associated with resistance to hormone therapy^19^. Methylation Beta values for cg15626350 in the ESR1 promoter varied significantly across BCCS subtypes in BRCA overall (*p* = 2.0 × 10^−52^, Fig. 7a) and restricted to ER+ tumors (*p* = 2.2 × 10^−43^). Beta values of methylation sites were also screened for their abilities to predict membership of individual BCCS subtypes (see the “Methods” section). The most significant predictions were for PCS1, NCS1, and NCS2; Fig. 7b–d displays the distributions for selected methylation sites. Promoter methylation of BCL2 (cg24408313) predicted membership in PCS1 with AUC 0.81 among ER+ tumors; loss of promoter methylation of TFF3 (cg04806409) predicted membership in NCS2 with AUC 0.92 among ER− tumors; and loss of promoter methylation in GFAP (cg21944455) predicted membership in NCS1 among ER− tumors with AUC 0.93. These results on the promoter methylation of ESR1 and TFF3 were concordant with those on their expression levels (Supplementary Fig. 3). Together these results showed significant variation in patterns of methylation across BCCS subtypes.

### Comparison of BCCS to TNBC subtyping systems

The relationships between BCCS subtypes for TNBC tumors and prior subtyping systems for TNBC, specifically TNBCtype^6^ and the Burstein subtypes^7^ were analyzed. The Burstein subtypes were derived from GSE76275 (see the “Methods” section). BCCS subtypes were computed for these samples using BCCSclassifier (ER−). The refinement of TNBCtype subtypes to the 4 TNBCtype4 subtypes^20^ was computed using the TNBCtype tool (see the “Methods” section). While GSE76275 reported 198 samples as TNBC, the TNBCtype tool computed subtypes only for the 157 samples it assessed as estrogen-receptor negative by ESR1 expression level.

Tabulation of the BCCS subtypes NCS1, NCS2, and NCS3 and the Burstein subtypes (Supplementary Table 8) showed 71% agreement between NCS2 and LAR, and 62% agreement between NCS3 and MES, while BLIS and BLIA largely refined NCS1. In fact, 83% of the pairs of samples clustered together by Burstein subtypes, were also clustered together by BCCS. The TNBCtype4 system also refined NCS1–3 in the sense that 77% of pairs of samples clustered together by TNBCtype4 were clustered together by BCCS. The Burstein subtypes and TNBCtype4 subtypes (Supplementary Table 9) showed a lesser degree of concordance. Within NCS1, only 19% of pairs were clustered by both Burstein subtypes and TNBCtype4. These results showed that both Burstein subtypes and TNBCtype4 refined BCCS, but did so in different ways.

### Differential expression of genes involved in response to CDK4/6 inhibitors

**Fig. 8:**
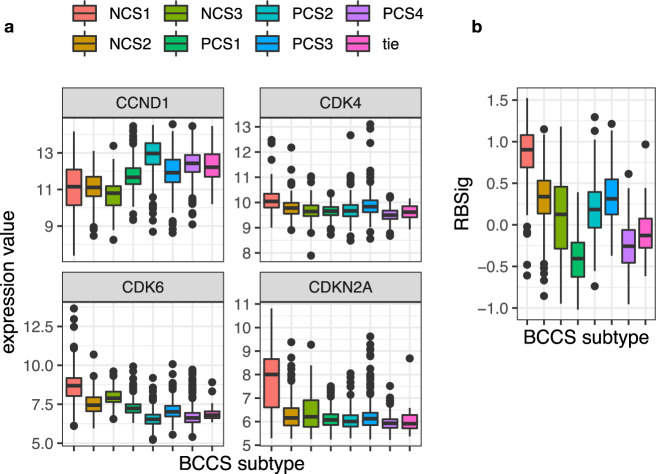
Distribution by BCCS subtypes in METABRIC for expression of genes related to CDK4/6 inhibitor activity, and the RBSig signature. **a** This figure plots distributions by BCCS subtypes in METABRIC of the expression levels of CCND1, CDK4, CDK6, and CDKN2A, which have been associated with CDK4/6 inhibitor activity. **b** This plots the degree of variation in values of the RBSig score by BCCS subtype of METABRIC. The RBSig score was created to measure the likelihood of resistance to CDK4/6 inhibitors. The midline in a boxplot indicates the median, the upper and lower edges indicate the quartiles, and the whisker lines are 1.5 times the interquartile range.

The CDK4/6 inhibitor class of drugs has been found to improve survival in metastatic ER+ breast cancer patients^21–25^ and is being evaluated as adjuvant treatment for early breast cancer patients^26^. Although single-gene biomarkers for CDK4/6 inhibitor sensitivity have not been identified, a positive response to this class of drugs has been associated with high expression of CCND1, moderate expression of the targets CDK4 and CDK6, and low expression of the inhibitor CDKN2A^26,27^. The RBSig genomic signature was developed to predict patients likely to be resistant to CDK4/6 inhibitors^28^. The ER+ patients most likely to be considered for adjuvant systemic therapy would be the poor prognosis patients in PCS2 and PCS3. We found that mean expression of CCND1 was significantly higher in PCS2 than in the remaining ER+ tumors (*p* < 10^−53^ in all cohorts) and RBSig score was significantly higher in PCS3 than PCS2 (*p* < 10^−50^ in all cohorts, see Fig. 8 for METABRIC results). In METABRIC ER+ samples, only 1/29 samples with a mutation in RB1 were in PCS1, but the remaining 28 were not disproportionately represented in PCS2–4 (*p* = 0.5). As expected, the RBSig score was significantly higher in RB1 mutated samples than RB1 wild-type samples (*p* = 1.7 × 10^−10^). Results in BRCA were similar.

## Discussion

Herein, we developed the Breast Cancer Consensus Subtype (BCCS) system based on whole-transcriptome data from fresh-frozen primary tumor samples. The derivation method was designed to reduce bias due to the clustering method or expression assay technology. The BCCS subtypes (four ER+, three ER−) exhibited differences in tumor biology as assessed by gene expression, patterns of DNA alteration, and immune and stromal cell infiltration. Features specific to some subtypes have been associated with treatment outcomes in other studies, but the clinical utility of BCCS remains to be established.

The most novel feature of the BCCS subtype system was the partition of poor prognosis ER+ tumors into two subtypes, PCS2, and PCS3. Both subtypes were enriched for pathways associated with a high proliferation rate, however, PCS3 (18% of ER+ tumors) was elevated in IFN-*γ*-signaling and cytotoxic lymphocyte infiltration compared to PCS2. These characteristics of PCS3 were previously identified in a set of tumors resistant to aromatase inhibitors^29^. Of note, methylation in the promoter of ESR1, which is being investigated as an indicator of hormone therapy resistance^30^, was significantly more common in PCS3 than PCS2.

Staining intensity for PD-L1 protein has been used to select metastatic cancer patients for treatment by ICI, however, treatment response in PD-L1+ tumors of multiple cancer types has been uneven^31^. Analysis of clinical trials for TNBC have shown improved response for PD-L1+ tumors with high TIL counts and infiltrates by CD8+ T-cells^32^. We have shown that in addition to high PD-L1 expression, NCS3 and PCS3 tumors had elevated IFN-*γ*-signaling and cytotoxic lymphocyte infiltration. PCS3 tumors also had high TMB, which has indicated a positive response to ICI in multiple cancer types^33^. These multiple indicators suggest that NCS3 and PCS3 tumors may be responsive to ICI, although data on ICI response is unavailable.

In addition to two poor prognoses ER+ tumors, BCCS presented two good prognoses ER+ subtypes, PCS1 and PCS4, that exhibited significantly different features with respect to gene expression, mutation frequency, copy number changes, tumor cellularity, and promoter methylation of ESR1. Of note, tumors with low cellularity and high stromal cell infiltration were most likely in PCS1. Single-cell analysis of TNBC samples showed that the TNBCtype subtypes with low cellularity (IM and MSL) were largely determined by gene expression from stromal and immune cell infiltrates^20^. This led investigators to conclude that IM and MSL were not distinct tumor subtypes, eliminating them from TNBCtype. In the case of PCS1, however, the majority of samples had PIK3CA mutations, exhibited gain in 1q, and many had promoter methylation for ESR1. These are features of tumor cells distinct from those of other subtypes, providing evidence that PCS1 is a distinct tumor subtype.

We chose to analyze ER+ and ER− samples separately because subtyping of all tumor samples (BCS1–5) separated them by ER status, a fortiori. This was not the case for HER2 status; HER2+ tumors were heterogeneous with respect to BCCS (Supplementary Table 5), having significant representation in PCS1–3 and NCS2–3. In fact, the differences in biological activity between PCS1–3 revealed by GSEA largely held in HER2+ and HER2− tumors alike (Supplementary Fig. 4). Previously, heterogeneity of HER2+ tumors with respect to TIL counts has been associated with differential treatment response^10–13,34^. The BCCS subtypes described heterogeneity in HER2+ tumors with respect to numerous traits beyond lymphocytic infiltration.

Among the limitations of BCCS, we note that all cohort samples were from primary tumors. The biology of metastases, especially in relation to the microenvironment, could be significantly different. Analyses of metastases will need to be carried out as an independent study. Also, gene expression in the study cohorts was obtained by whole-transcriptome assay of fresh-frozen tissue. Clinical application of BCCS will require an analytically validated test that uses formalin-fixed paraffin-embedded (FFPE) tissue and a specific assay technology, which is beyond the scope of this initial study.

A further limitation of the BCCSclassifier is that it is necessary to determine the ER status of a tumor prior to classification. For some patients with a low percentage of ER-positive staining, the ER status may be uncertain^35^, leading to uncertainty in the BCCS classification. Secondly, some tumors may have a mixed subtype; i.e., features of multiple subtypes. This could be due to intra-tumoral heterogeneity, as occurs in colon cancer^36^. The use of continuous scores to estimate the prevalence of a BCCS subtype among the cells in a tumor would reduce the impacts of these limitations, as previously published for colorectal cancers^37^. The derivation of continuous scores for BCCS prediction is beyond the scope of this paper.

In conclusion, we developed BCCS using whole-transcriptome data from fresh-frozen primary tumor samples, having biological and clinical features independent of cohort and gene expression assay platform. Among ER+ tumors, BCCS identified two poor prognosis subtypes, which differed in the degree of immune activity, and two good prognosis subtypes with one high in stromal infiltration and the second elevated in hormone response. The BCCS subtypes described significant heterogeneity in HER2+ tumors. Clinical application of BCCS will require an analytically valid assay applicable to FFPE tissue and evidence that subtypes predict responses to treatments.

## Methods

### Study cohorts

The BCCS were discovered and analyzed using the following three publicly available whole-genome transcription datasets.

METABRIC cohort (*n* = 1992). The METABRIC patient cohort was formed from 1992 primary breast cancer patients^14^. Clinico-pathological traits (Supplementary Table 1) were supplemented with data on distant metastasis-free survival (DMFS) provided by METABRIC investigators (unpublished). Gene expression values for tumor samples from METABRIC patients, measured with IlluminaHuman-v3 beadarray, were obtained from European Genome-phenome Archive (EGAD00010000210, EGAD00010000211). Data for DNA copy number alterations were obtained from EGAD00010000213 and EGAD00010000215. Mutation data for a selected set of 173 genes for METABRIC samples were obtained from cBioPortal^38^. We partitioned METABRIC into two cohorts, METABRIC-A (*n* = 997) and METABRIC-B (*n* = 995) consisting of the previously published^14^ discovery and validation cohorts. To denote estrogen receptor status we used the status determined by gene expression as reported by the authors.

Affymetrix cohort (*n* = 2923). This cohort was formed by merging clinico-pathological data (Supplementary Table 1) and gene expression data (assessed with the hgu133a array) from the following cohorts (the number of samples in in parentheses): GSE25065 (198), GSE256055 (310), GSE20194 (278), GSE20271 (177), GSE1456 (159), GSE22093 (103), GSE23988 (61), GSE42822 (90), GSE3494 (251), GSE7390 (198), GSE12093 (136), GSE6532 (178), GSE2034 (286), GSE11121 (200), GSE17705 (298). Raw CEL file data was normalized with fRMA (frozen RMA)^39^ and corrected for batch effects with ComBat using the SVA R package.

BRCA cohort (*n* = 1035). Clinico-pathological data for the TCGA-BRCA cohort^40^ (BRCA) was obtained from NCI Genomic Data Commons (GDC, https://gdc.cancer.gov) and Broad Institute GDAC Firehose (https://gdac.broadinstitute.org) and summarized in Supplementary Table 1. PAM50 subtype assignments were obtained from the TCGAbiolinks R package. The BAM files produced by RNA-sequencing were obtained from GDC and aligned to the Ensembl v90 genome using Rsubread^41^. Transcript per million (TPM) expression measures were computed with the Rsubread feature counts algorithm^42^. Unless otherwise noted, log2(TPM + 1) was used as the expression value of a gene for a sample in this cohort. For GSEA, described below, gene expression measures were computed using the DESeq2 R package^43^, as recommended by GSEA authors. Estrogen receptor status was determined by IHC. Somatic mutation data for BRCA was obtained from LinkedOmics (http://www.linkedomics.org/data_download/TCGA-BRCA/). Tumor mutation burden was computed using the maftools R package^44^. DNA methylation data for BRCA samples were downloaded from GDC. Methylation data from the Illumina Human Methylation 450 arrays were restricted to the probes in common with the Illumina Human Methylation 27 array. Tumor purity (aberrant cell fraction) has been used as a measure of tumor cellularity. Tumor purity computed with ASCAT^45^ was obtained from COSMIC (https://cancer.sanger.ac.uk/cosmic/).

### Compliance with ethical requirements

Data acquired in the METABRIC study were obtained under the ethical requirements of the relevant institutions and the study group^14^. The collection of human data for TCGA-BRCA was carried out under the requirements of the TCGA Network^40^. All other data used in this study were obtained from the Gene Expression Omnibus and subject to the National Center for Biotechnology Information requirement that appropriate consent/permission had been obtained to submit data to a public repository. All ethical requirements from the use of the above human data were followed in this study.

### Representation of gene expression

In this study, analysis was restricted to genes, as represented by ENTREZIDs, whose expression was measured in each of the above three cohorts. In the METABRIC cohort, we associate with each such gene the Illumina probe annotated to this gene with maximal interquartile range in the METABRIC-A cohort. The expression of the gene for a sample in METABRIC is then reported as the expression level of the selected probe. In the Affymetrix cohort, the jetset R package was used to select a probe to represent a gene. We further restricted to the ENTREZIDs associated with a unique gene in the BRCA gene count data from Ensembl v90. In the end, for each of a set of 11,569 ENTREZIDs and each sample in any of the three study cohorts, there is a unique expression value of the ENTREZID for the sample.

### Consensus subtyping method

The consensus subtyping method, as it was applied to create the CMS of colorectal cancer^9^, consists of the steps: (1) creating multiple subtyping systems using a variety of methods, (2) clustering the network of all possible subtypes, (3) for each clustered set of subtypes, identifying samples frequently assigned to the cluster’s subtypes, so-called core samples. These steps, as applied in this study, are described in the following subsections. This method was applied in the METABRIC-A cohort for subtyping all breast cancer samples, the METABRIC ER− samples for subtypes specific to ER- breast cancer, and the METABRIC ER+ samples for subtypes specific to ER+ breast cancer.

### Independent subtyping methods

Six independent methods (A–F) were applied to the training domain to create different subtyping systems. Each of the six methods involved filtering the genes considered, applying an unsupervised clustering method to the filtered gene expression dataset, and selecting an optimal number of clusters (summarized in Supplementary Table 10).

Methods A–C were executed using the ConsensusClusterPlus R package^46^, with different gene filtering methods and unsupervised clustering methods. ConsensusClusterPlus uses resampling to increase the robustness of the resulting clusters, allowing resampling on the genes, the samples, or both. The package also enables the selection of an optimal number of clusters using several quality metrics. ConsensusClusterPlus produces a *consensus matrix* that measures the fraction of times the sample pair determined by row and column indices were clustered together in the resampled classifiers. Such a matrix exists for each possible number of clusters. To select an optimal number of clusters, ConsensusClusterPlus provides three analyses of the consensus matrix for each number *k* of clusters. First, a heatmap plot of the matrix illustrates the degree of coherence of the *k* clusters. Second, the cumulative distribution function (CDF) of the consensus matrix for *k* clusters is a more quantitative measure of cluster coherence. An optimal number of clusters by this metric is the value at which the area under the corresponding CDF has reached a plateau. Finally, ConsensusClusterPlus produces a “delta plot” to show the amount of change in area under the CDF between *k* and *k* + 1 clusters. In all methods we considered the range of 2–8 clusters.

#### Method A

In this method, the set of genes was filtered to those with median absolute deviation (MAD) >0.5. (The median absolute deviation of a vector of numbers *v* = *v*_1_,…,*v*_*m*_ is median(|*v*_*i*_–median(*v*)|).) The clustering method selected was hierarchical clustering with average linkage and distance 1−Pearson correlation of the vectors of gene expression values. Clustering was executed with ConsensusClusterPlus, resampling 90% of the *genes* 1000 times, and considering 2–8 clusters.

#### Method B

For Method B, the genes were ranked by variance and the highest ranked 15% were selected for the distance measure. The clustering method selected was hierarchical clustering with ward linkage and distance 1−Pearson correlation of the vectors of gene expression values. Clustering was executed with ConsensusClusterPlus, resampling 90% of the *samples* 1000 times and considering 2–8 clusters.

#### Method C

In this method, from among the genes having variance test *p*-value < 0.01, select those with the highest 10% coefficients of variation. The clustering method was the same as in Method B; i.e., hierarchical clustering with ward linkage. Clustering was executed with ConsensusClusterPlus, resampling 90% of both samples *and* genes 1000 times, considering 2–8 clusters.

In Methods A–C the clustering was carried out in the framework of the ConsensusClusterPlus package. For Method D, we used Partition Around Medoids (PAM)^47^ and for E and F we used nonnegative matrix factorization^48^.

#### Method D

The set of genes was filtered to those with the highest 5% of interquartile ranges. Clustering was performed by the Partition Around Medoids (PAM) method, testing 2–8 possible clusters. The gap statistic^49^ was used to select the optimal number of clusters. These steps were carried out using functions from the cluster R package^50^.

#### Method E

Possible sets of genes were tested by restricting to those with standard deviation >0.5, 0.8, 1, or 1.1 for the expression values in the training domain. Clustering was performed for each expression dataset with non-negative matrix factorization as implemented in the NMF R package^51^. The NMF procedure was run 30 times, testing 2 through 8 possible clusters. The number of clusters and set of genes was selected using heatmap and cophenetic coefficient diagnostic plots.

#### Method F

The set of genes was filtered to those with an interquartile range >1.2. Otherwise, Method F agreed with Method E.

### Network analysis of subtype association

This analysis followed the process described in Guinney et al.^9^. In our study, the above six methods were applied to the training domain of samples (METABRIC-A, METABRIC ER+, METABRIC ER−), for each of the three subtyping projects. Application of the above six methods to the training domain resulted in a set of *k* total subtypes, with a different *k* for each of the three projects. A pair of subtypes were considered significantly connected if a hypergeometric test for over-representation of samples in the intersection of the subtypes had Benjamini–Hochberg adjusted *p*-value < 0.001. A graph network was defined with nodes these *k* subtypes, and with an edge between a pair of nodes if they were significantly connected. To cluster such a network, resulting in groups of associated subtypes, Markov cluster analysis was applied with the MCL R package^52^. To assess confidence in the robustness of such an association, the MCL cluster algorithm was repeated 1000 times, each iteration sampling 80% of the samples. A *k* × *k* matrix was defined with each entry the frequency that the pair of subtypes were in the same cluster over the 1000 applications of MCL. These frequencies were compared to the cluster assignments by MCL applied to the network of all samples to measure the stability of a subtype’s cluster assignment. Specifically, for a subtype assigned to a cluster, the stability score of the subtype was the average frequency for all pairs formed by this subtype and other subtypes in the cluster. Clustering performance was evaluated with weighted silhouette width using the WeightedCluster R package, using the stability scores as weights.

An application of MCL can result in varying numbers of clusters depending on the inflation factor parameter. MCL was applied for inflation factors from 1 to 10, in increments of 0.25. The MCL result with the lowest inflation factor at which the resulting weighted silhouette width reaches a maximal plateau was chosen, under the constraint that no cluster had fewer than 50 core samples (see the next subsection).

### Identification of core subtype samples

The preceding process resulted in clusters of subtypes. Each cluster gives rise to a subtype of the training domain as follows. For such a sample *x* in the training domain and a cluster C consisting of subtypes *s*_1_,…,*s*_*k*_, a hypergeometric test for over-representation of *x* as a member of the s_i_’s was performed. Then *x* was a core sample of C if the hypergeometric test had a *p*-value < 0.05. The core samples of all clusters formed the Consensus Training Subtypes for the subtyping project.

### BCCSclassifier subtyping algorithm

The above consensus subtyping method is too lengthy to repeat in practical applications, and cannot be applied as a single-sample classifier. Instead, a computer application to generate subtypes on an arbitrary whole-transcriptome dataset was trained on the Consensus Training Subtypes of the training domain. The training domain was further partitioned randomly into discovery set (75%) and test set (25%). Candidate classification applications were developed on the discovery set, and the optimal performer on the test set was selected.

The core of this application is a set of prediction models derived with kTSP^53^. A kTSP classifier contains a set of pairs of genes, and for a sample to be classified, it tests which gene in each pair has the highest expression and makes a classification decision based on the results of these tests. A single kTSP classifier decides whether a sample is more appropriately classified into subtype A or subtype B. To generalize the method to an arbitrary number of subtypes, *C*_1_,…,*C*_*n*_, a kTSP classifier is defined for each pair of subtypes. Then, to classify a new sample, the kTSP classifiers for all pairs are evaluated, and the sample is classified into the subtype that is most frequently predicted, if one exists, and is labeled a “tie” if none exists. Here, kTSP predictors were derived using the ktspair R package.

In typical applications, kTSP classifiers reach optimal performance with fewer than 10 pairs of genes. To add information from other genes we used a resampling method to derive what we have called a Multi-kTSP classifier. Specifically, we formed a family of m subsets of genes, for some predetermined number m, each consisting of randomly sampled 75% of the overall set of candidate genes. For each of *m* sets of genes, we derived an optimal kTSP classifier from this set of genes. The Multi-kTSP classifier consisted of the resulting set of *m* kTSP classifiers. To classify a new sample, we evaluated each of the *m* predictors, and assigned the sample to the most frequently predicted subtype, or “tie” if there wasn’t a unique maximum. We created such predictors by selecting genes from among the most highly varying 1500 genes, and creating 250, 500, or 750 kTSP models in the discovery subset of training domain. We selected the minimal number of models for which accuracy in the test set as a predictor of the Consensus Training Subtypes reached a plateau.

### Alternative subtyping methods

To test the robustness of the subtyping method we selected an alternative set of six methods for re-subtyping the ER+ METABRIC dataset. The six methods varied in how the set of genes was filtered, how distance between samples was computed from expression data, and the method of clustering (Supplementary Table 11). Only methods that produced 3–8 subtypes with more than 50 samples were adopted. Of note, fuzzy clustering (Method III) was used by Jézéquel et al.^8^, non-negative matrix factorization (Method II) was used by Burstein et al.^7^, and *k*-means and consensus clustering (similar to Method I) was used by Lehmann et al.^6^.

### Gene set enrichment analysis

GSEA^54^ was performed on each cohort using the fgsea R package. As a measure of significance, we used an adjusted *p*-value, as reported by the package. Enrichment of each of the Hallmark v7 gene sets (https://www.gsea-msigdb.org/gsea/msigdb/collections.jsp) was tested. For analysis of BRCA, gene expression values normalized by DESeq2 were used^43^, as recommended by the GSEA authors.

### Microenvironment Cell Populations counter (MCPcounter)

The MCPcounter system^55^, implemented as an R package (http://github.com/ebecht/MCPcounter), was used to quantify the abundance of populations of immune and stromal cells in a tumor sample. For each population of cells, and each sample, MCPcounter computes the mean of expression levels of a population-specific set of genes for this sample. These population means were used as a measure of the infiltration of the cell population in this sample. To create measures on a uniform scale, population means were median centered.

### Analysis of BCCS with DNA methylation

Differential methylation of specific sites with respect to BCCS subtypes was tested in BRCA. Specifically, for each subtype and array probe we applied the Kruskal–Wallis tested to the probe’s Beta values and membership in the subtype, and then ranked the probes by significance.

### TNBC samples and subtypes

Subtype systems for TNBC were analyzed in the GSE76275 TNBC cohort (*n* = 198). This dataset was formed from GSE76275 samples reported by the authors as triple negative^7^. Gene expression values were computed by fRMA applied to raw CEL files from Affymetrix hgu133plus2 arrays. The six subtypes of TNBC developed by Lehmann et al.^6^ were subsequently computed with the TNBCtype algorithm^56^. We applied this algorithm through the web interface (https://cbc.app.vumc.org/tnbc/). TNBCtype4 subtype of a sample was computed from the six subtype probabilities^20^.

### Statistical methods

All statistical analyses were performed using R (http://www.r-project.org) version 4.0 and Bioconductor packages (http://bioconductor.org) version 3.13. Kaplan–Meier survival models were plotted with the *survminer* R package. The result of a statistical test was considered significant if the *p*-value was < 0.05 unless an exception was explicitly stated. Hypergeometric tests were performed with the dhyper and phyper functions. To compare the mean values of a continuous variable between subsets we used the non-parametric Kruskal–Wallis test (two-sided). Accuracy and Cohen’s kappa statistic were used to assess the significance of a discrete predictor of subtypes. The midline in a boxplot indicates the median, the upper and lower edges indicate the quartiles, and the whisker lines are 1.5 times the interquartile range. The ability of a continuous score to predict membership in a subtype was assessed with the receiver operator characteristic (ROC) curve. The area under the ROC curve (AUC) gives a numerical measure of a score’s predictive significance. The quality of the prediction is better than random if AUC is >0.5 and improves as AUC increases up to 1. The *plotROC* R package was used for these computations.

### Reporting summary

Further information on research design is available in the Nature Research Reporting Summary linked to this article.

## Supplementary information

Supplementary Information

Supplementary Data 1

Reporting Summary

## Data Availability

The Affymetrix study cohort was formed from samples from the following cohorts which can be found at National Center for Biotechnology Information, U.S. National Library of Medicine (https://www.ncbi.nlm.nih.gov/gds/): GSE25065, GSE256055, GSE20194, GSE20271, GSE1456, GSE22093, GSE23988, GSE42822, GSE3494, GSE7390, GSE12093, GSE6532, GSE2034, GSE11121, GSE17705. Data for additional TNBC samples were obtained from GSE76275. Data for the TCGA-BRCA project is available as follows: gene expression data from NCI Genomic Data Commons (GDC, https://gdc.cancer.gov); clinical and pathological data from GDC, Broad Institute GDAC Firehose (https://gdac.broadinstitute.org), and TCGAbiolinks R package (https://bioconductor.org/packages/release/bioc/html/TCGAbiolinks.html); somatic mutation data from LinkedOmics (http://www.linkedomics.org/data_download/TCGA-BRCA/). Gene expression and copy number data for METABRIC are available from European Genome-phenome Archive (https://ega-archive.org) as EGAD00010000210, EGAD00010000211, EGAD00010000213, EGAD00010000215. Mutation data for selected genes from METABRIC can be obtained from cBioPortal (https://www.cbioportal.org). The BCCS subtype assignments for all study samples are available as Supplementary Data 1. Data on assignments of samples into training and validation sets can be obtained by reasonable request from the corresponding author.

## Code availability

BCCSclassifier software is available as an R package from https://github.com/sbuechler/BCCSclassifier. It is distributed under a Creative Commons license that permits free unrestricted use for non-commercial purposes.
