# Supplementary material for: Breast Cancer Consensus Subtypes: A system for subtyping breast cancer tumors based on gene expression
Source: NPJ Breast Cancer. 2021 Oct 12;7:136. doi: 10.1038/s41523-021-00345-2 (PMC8511026; doi:10.1038/s41523-021-00345-2)
Supplement: Supplementary file 1 — Supplementary Information [file 41523_2021_345_MOESM1_ESM.pdf]

Breast Cancer Consensus Subtypes: A system for subtyping breast cancer tumors based on gene expression

Christina Horr, Steven A Buechler

SUPPLEMENTARY FIGURES

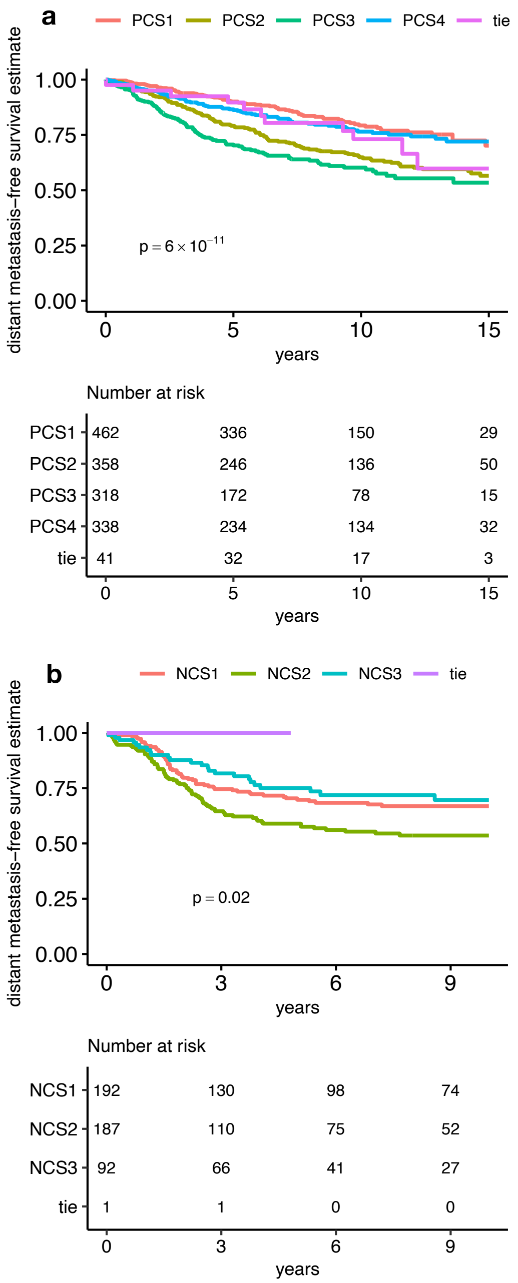

Supplementary Figure 1. Kaplan-Meier plots for BCCS subtypes in METABRIC cohort. Distant metastasis-free survival proportions were estimated for (a) METABRIC ER+ samples, and (b) METABRIC ER- samples

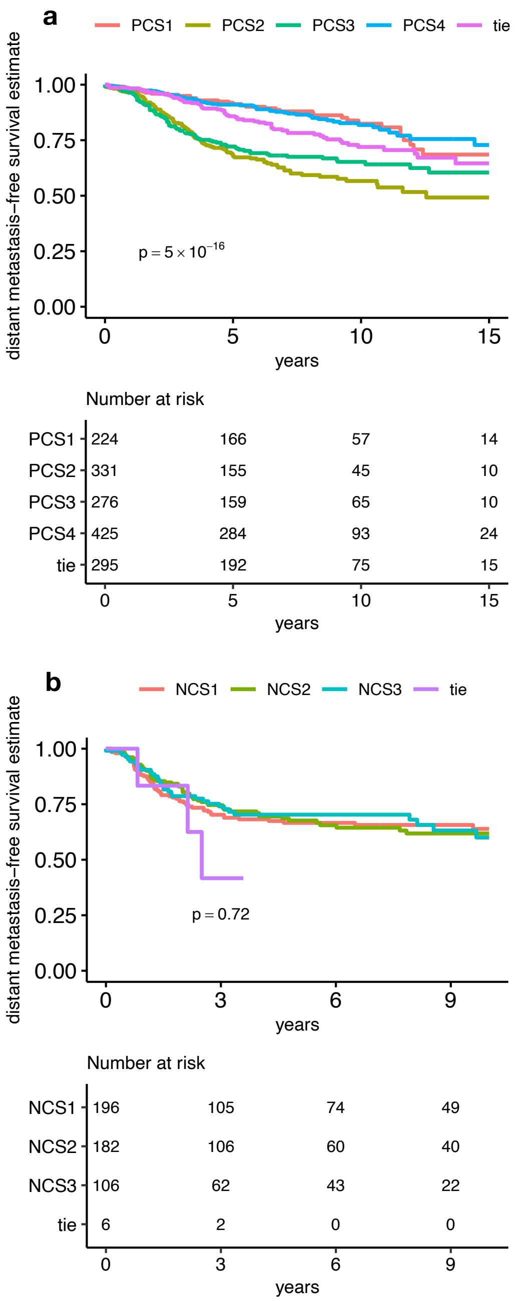

Supplementary Figure 2. Kaplan-Meier plots for BCCS subtypes in Affymetrix cohort. Distant metastasis-free survival proportions were estimated for (a) Affymetrix ER+ samples, and (b) Affymetrix ER- samples.

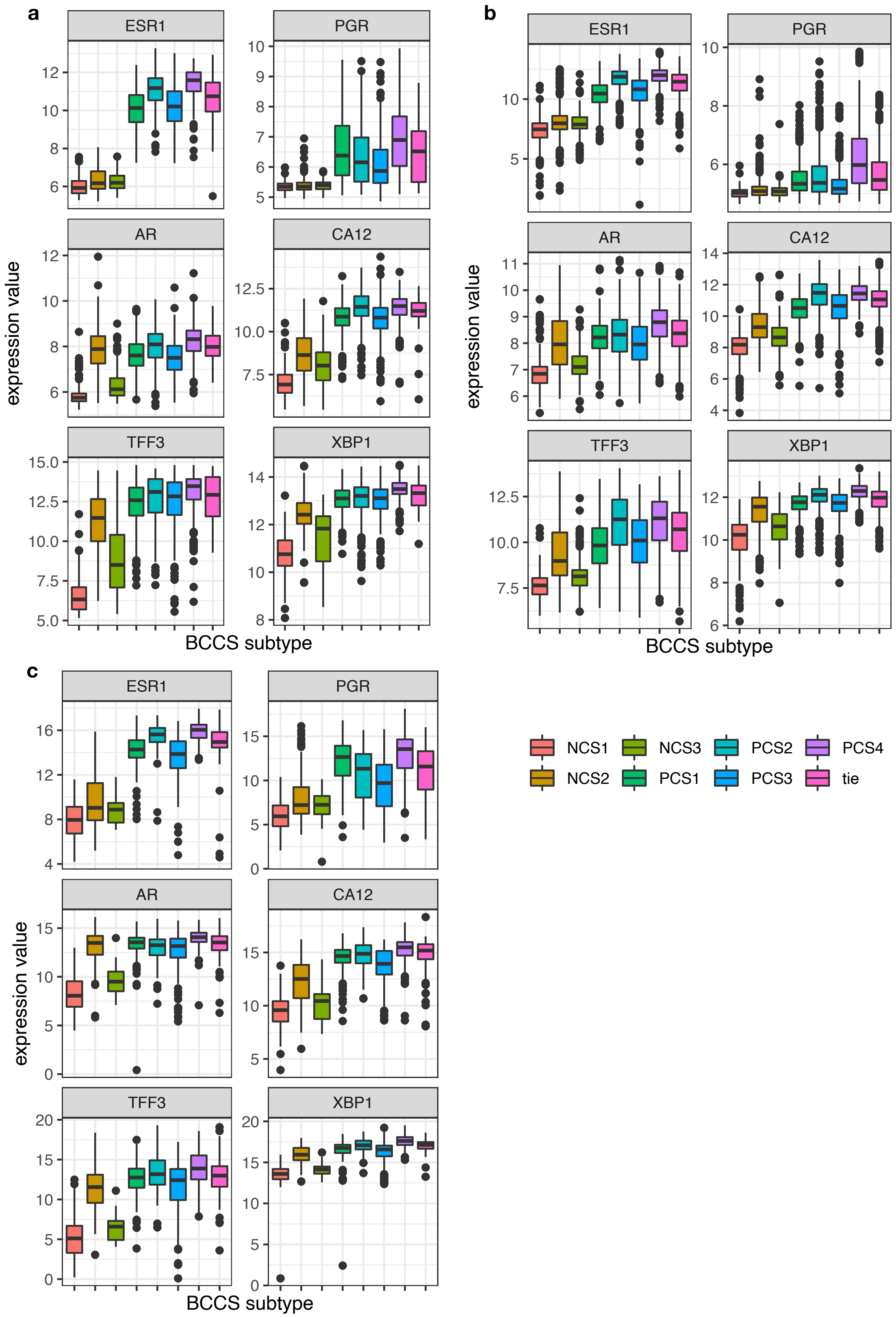

Supplementary Figure 3. Distribution of expression of selected hormone response genes with respect to BCCS. Distribution with respect to BCCS for expression of ESR1, PGR, AR, CA12, TFF3, XBP1 are plotted for (a) METABRIC, (b) Affymetrix, and (c) BRCA. The midline in the boxplot indicates the median, the upper and lower edges indicate the quartiles, and the whisker lines 1.5 times the interquartile range.

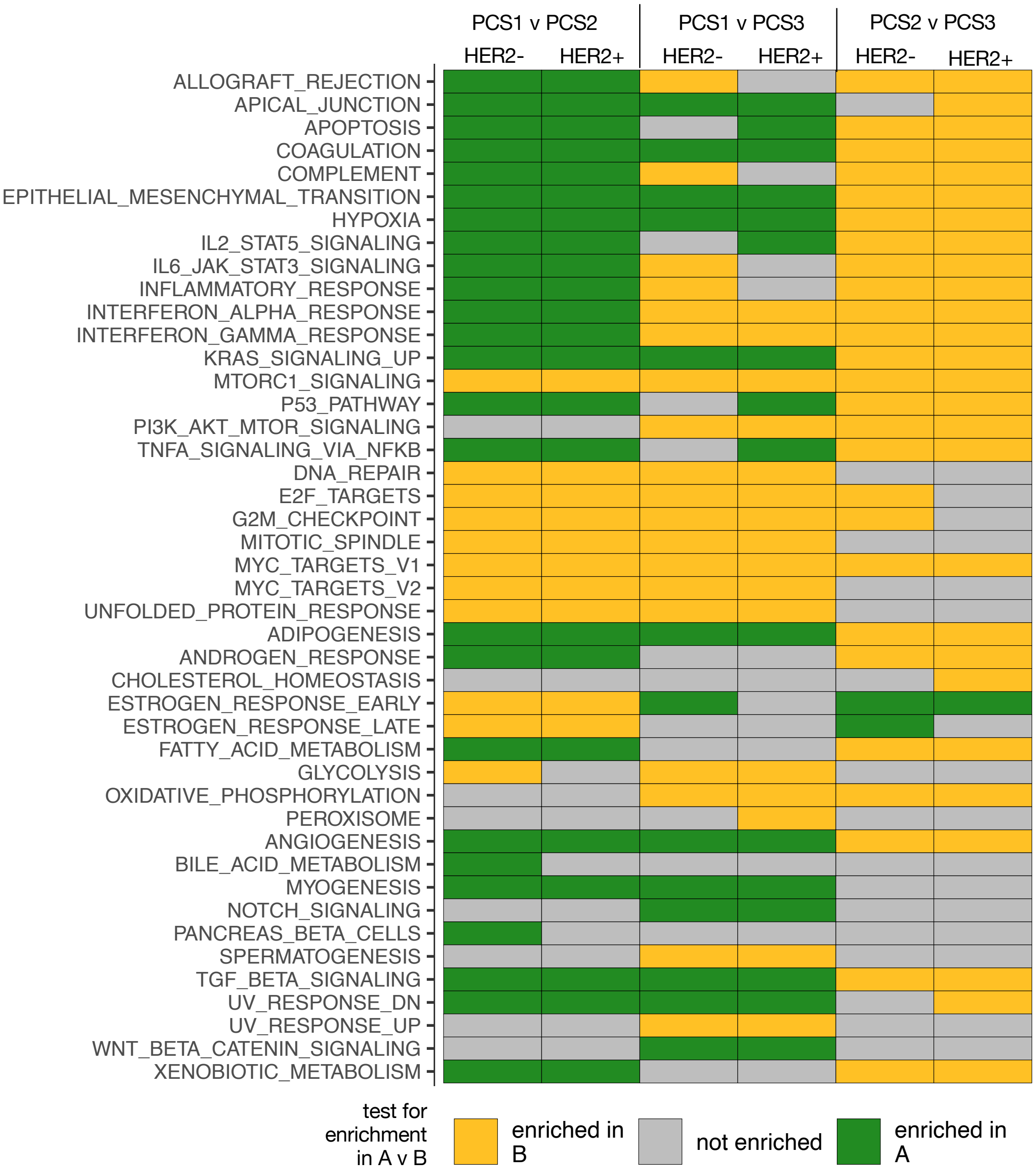

Supplementary Figure 4. Gene set enrichment by GSEA for pairs of PCS1-PCS3 subtypes of METABRIC restricted to HER2+ and HER2- tumors. We tested each Hallmark gene set that was reported in Figure 3. A gene set was considered enriched between a pair of subtypes if the adjusted p-value was < 0.05 in METABRIC. A test for enrichment in A versus(v) B reported the normalized enrichment score (NES); NES was positive if expression was enriched in A compared to B, and NES was negative if expression was enriched in B compared to A. A tile is colored green (respectively, goldenrod) for the given pair and gene set, if the adjusted p-value was < 0.05 and NES was positive (respectively, negative); a tile is gray if the adjusted p-value was ≥ 0.05 in METABRIC.

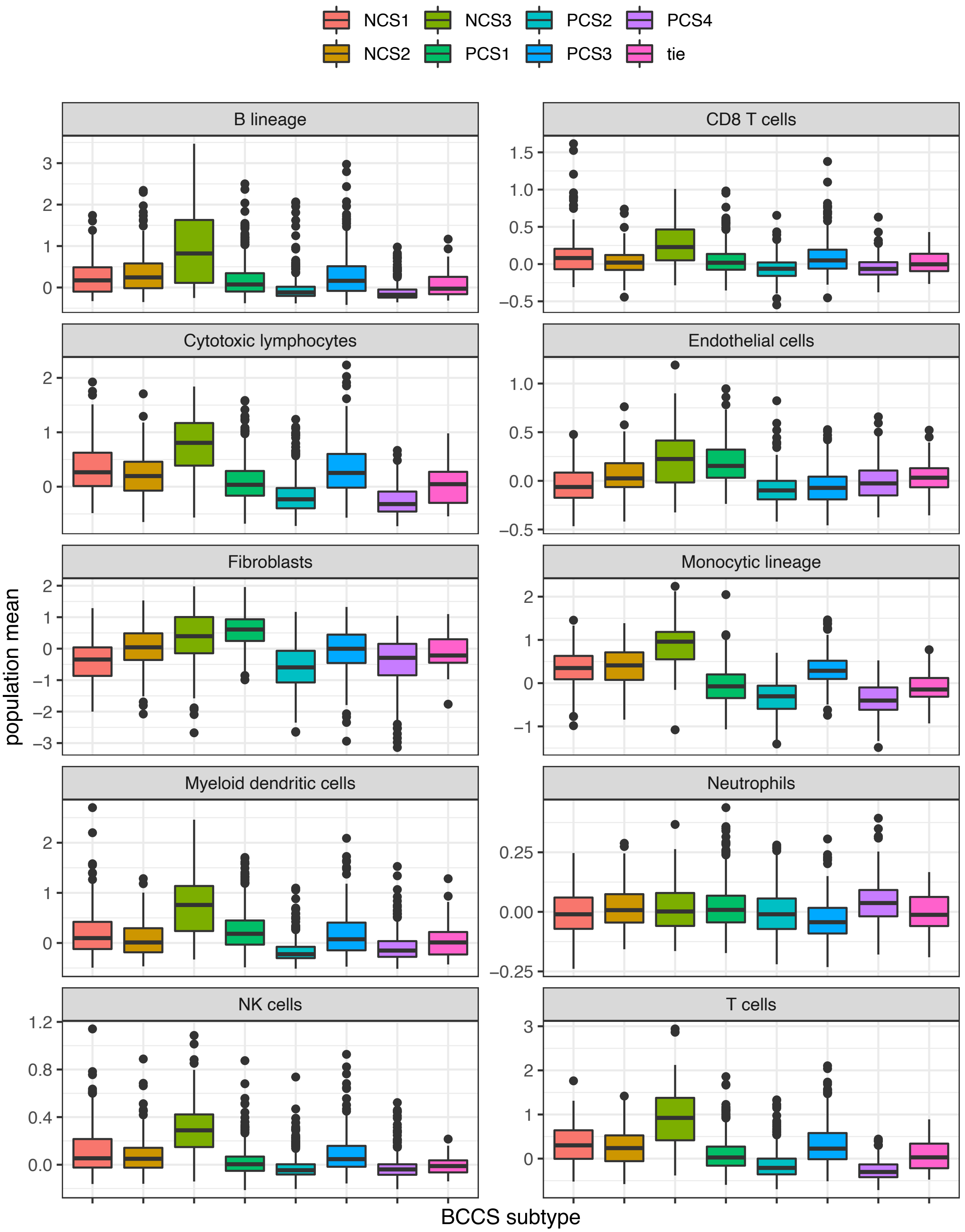

Supplementary Figure 5. Distribution with respect to BCCS subtypes of METABRIC for the population means for immune and stromal cell populations. The degree of infiltration by a cell population was assessed with the median-centered MCPcounter population means for B lineage, CD8 T cells, cytotoxic lymphocytes, endothelial cells, fibroblasts, monocytic lineage, myeloid dendritic cells, neutrophils, NK cells, and T cells. The midline in the boxplot indicates the median, the upper and lower edges indicate the quartiles, and the whisker lines 1.5 times the interquartile range.

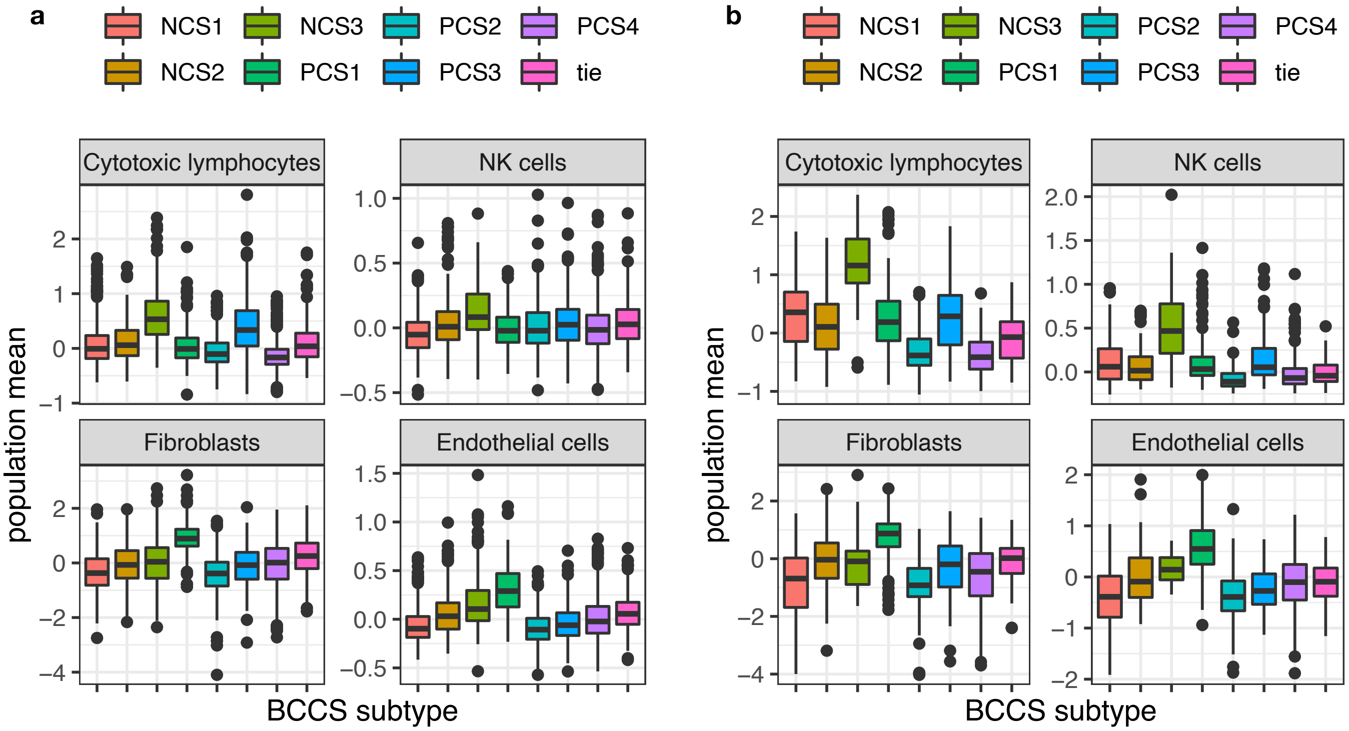

Supplementary Figure 6. Distribution by BCCS subtypes of measures of infiltration of selected immune and stromal cell populations. This figure plots the distributions by BCCS subtypes for the degrees of infiltration by cytotoxic lymphocytes, NK cells, fibroblasts and endothelial cells in (a) Affymetrix cohort, and (b) BRCA cohort. The degree of infiltration by a cell population was assessed with the median-centered MCPcounter population means. The midline in the boxplot indicates the median, the upper and lower edges indicate the quartiles, and the whisker lines 1.5 times the interquartile range.

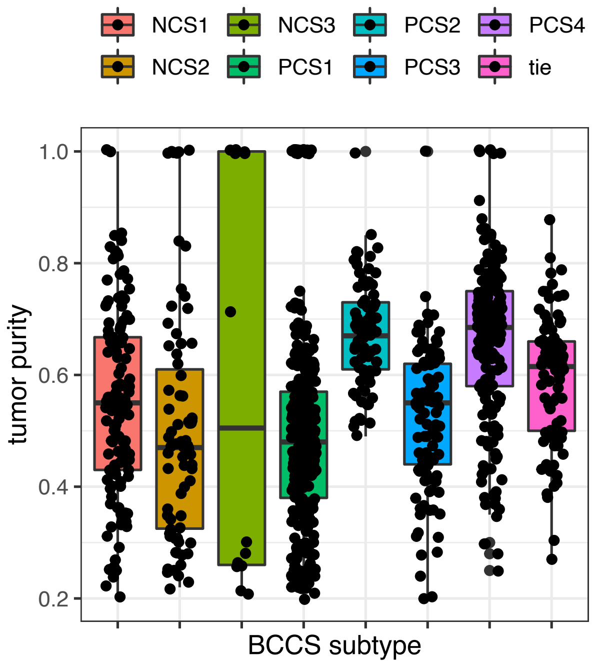

Supplementary Figure 7. Distribution of ASCAT tumor purity with respect to BCCS subtypes in BRCA. Values for individual points were plotted to inform the distribution of purity values for NCS3. The midline in the boxplot indicates the median, the upper and lower edges indicate the quartiles, and the whisker lines 1.5 times the interquartile range.

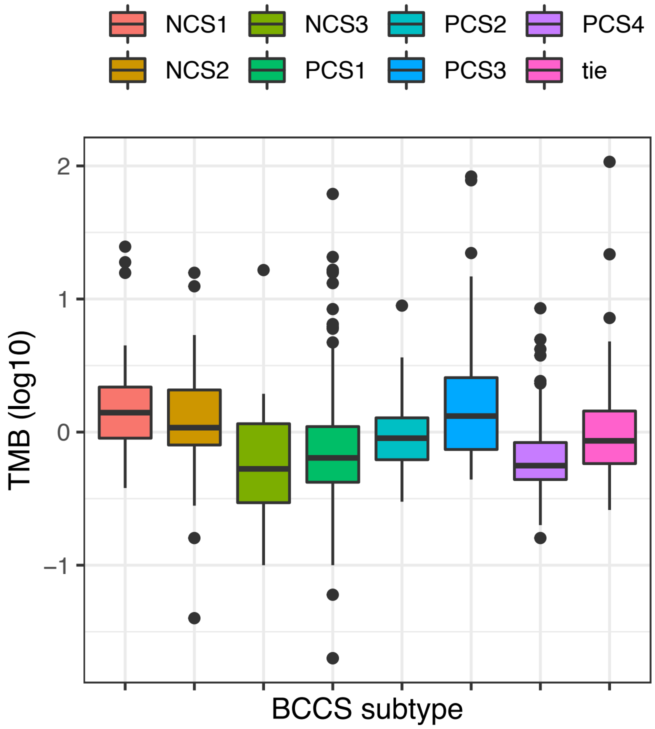

Supplementary Figure 8. Distribution of tumor mutation burden (TMB) with respect to BCCS subtypes in BRCA. The midline in the boxplot indicates the median, the upper and lower edges indicate the quartiles, and the whisker lines 1.5 times the interquartile range.

SUPPLEMENTARY TABLES

Supplementary Table 1. Patient cohorts analyzed

	METABRIC-A[1] (n = 997)	METABRIC-B[1] (n = 995)	Affymetrix (n = 2,923)	BRCA (n = 1,035)
ER status				
positive	798	720	2,062	799
negative	199	275	861	236
PR status				
positive	525	524	677	690
negative	472	471	621	340
NA	0	0	1,625	5
LN				
positive	483	467	958	548
negative	514	528	1,611	487
NA	0	0	354	0
HER2 status				
positive	215	226	125	196
negative	779	767	631	688
NA or equivocal	3	2	2,167	151
stage				
1	185	187	-	175
2	280	299	-	587
3	43	47	-	238
4	9	1	-	18
NA	480	461	2,923	17
grade				
1	72	98	245	-
2	415	360	895	-
3	510	447	886	-
NA	0	90	897	1,035

[1]The METABRIC cohort was partitioned into METABRIC-A and METABRIC-B cohorts

Supplementary Table 2. Distribution of samples in BC Consensus Training Subtypes T0-T5 of METABRIC-A cohort (n = 997) by ER status

	ER-	ER+
T0	5	87
T1	87	14
T2	97	12
T3	2	221
T4	0	289
T5	8	175

Supplementary Table 3. Numbers of samples and the proportion of samples in each BCCS subtype and each cohort

BCCS subtype	Affymetrix	BRCA	METABRIC
NCS1	296 (0.10)	140 (0.14)	193 (0.10)
NCS2	354 (0.12)	80 (0.08)	188 (0.09)
NCS3	201 (0.07)	16 (0.02)	92 (0.05)
PCS1	280 (0.10)	306 (0.30)	462 (0.23)
PCS2	463 (0.16)	87 (0.08)	358 (0.18)
PCS3	367 (0.13)	115 (0.11)	319 (0.16)
PCS4	555 (0.19)	199 (0.19)	338 (0.17)
tie	407 (0.13)	92 (0.08)	42 (0.02)

Supplementary Table 4. Distributions of clinical features in the BCCS subtypes for METABRIC and BRCA[1]

trait	cohort	NCS1	NCS2	NCS3	PCS1	PCS2	PCS3	PCS4	tie
n	METABRIC	193	188	92	462	358	319	338	42
	BRCA	140	80	16	306	87	115	199	92
LN+	METABRIC	0.51	0.62	0.45	0.43	0.43	0.57	0.41	0.45
	BRCA	0.35	0.56	0.44	0.54	0.59	0.6	0.54	0.6
size ≥ 2cm	METABRIC	0.64	0.62	0.42	0.45	0.61	0.63	0.58	0.54
	BRCA	0.76	0.78	0.81	0.67	0.82	0.85	0.71	0.78
PR+	METABRIC	0.03	0.05	0.05	0.73	0.62	0.54	0.79	0.67
	BRCA	0.04	0.1	0.13	0.91	0.83	0.68	0.9	0.78
age > 50	METABRIC	0.53	0.65	0.74	0.76	0.87	0.83	0.92	0.81
	BRCA	0.62	0.78	0.75	0.67	0.8	0.73	0.88	0.71
HER2+	METABRIC	0.08	0.7	0.27	0.09	0.24	0.34	0.07	0.17
	BRCA	0.08	0.41	0	0.17	0.18	0.35	0.11	0.26
grade ≥ 3	METABRIC	0.94	0.8	0.76	0.23	0.51	0.69	0.2	0.44
	BRCA	NA	NA	NA	NA	NA	NA	NA	NA
lobular histology	METABRIC	0.02	0.06	0.10	0.27	0.08	0.05	0.11	0.19
	BRCA	0.01	0.08	0	0.43	0.07	0.04	0.18	0.14

[1] Entries for n are the number of samples in the category and other entries are the proportion of samples in the subtype with the specified trait.

Supplementary Table 5. Distribution in BCCS subtypes of HER2 amplification status in METABRIC and HER2 status by IHC and FISH in BRCA

	METABRIC			BRCA[1]			
	HER2 gain	HER2 no gain	NA	HER2+	HER2-	E	NA
NCS1	15	178	0	11	114	4	11
NCS2	132	56	0	33	31	4	12
NCS3	25	67	0	0	14	1	1
PCS1	43	417	2	51	217	15	23
PCS2	86	271	1	16	56	9	6
PCS3	109	209	1	40	57	10	8
PCS4	24	314	0	21	142	13	23
tie	7	34	1	24	57	1	10

[1] HER2 status determined using data on HER2 IHC scores, copy numbers by FISH and HER2-cent17 ratio following CAP guidelines (https://documents.cap.org/documents/asco-cap-her2-recommendations.pdf); E = equivocal status

Supplementary Table 6. Relationship between BCCS subtypes and tumor cellularity in METABRIC cohort

	cellularity			
	high	moderate	low	NA
NCS1	124	49	19	0
NCS2	96	57	24	2
NCS3	24	38	22	0
PCS1	151	207	79	2
PCS2	210	130	13	0
PCS3	189	103	19	3
PCS4	164	134	33	1
tie	15	20	7	0

Supplementary Table 7. Rate of copy number alteration in BCCS subtypes of BRCA for alterations representative of integrative clusters[1]

	NCS1	NCS2	NCS3	PCS1	PCS2	PCS3	PCS4	tie
16q23.1 LOSS	0.19	0.19	0.19	0.28	0.47	0.27	0.62	0.40
17q12 GAIN	0.11	0.48	0.06	0.10	0.34	0.42	0.09	0.33
1q41 GAIN	0.53	0.35	0.06	0.45	0.55	0.57	0.68	0.54
5q11.2 LOSS	0.46	0.19	0.06	0.06	0.13	0.17	0.08	0.15
8p12 LOSS	0.30	0.34	0.19	0.17	0.73	0.40	0.28	0.54
8q24.21 GAIN	0.76	0.49	0.25	0.23	0.75	0.70	0.23	0.65

[1] IntClust 8 (16q23.1 LOSS, 1q41 GAIN), IntClust 3 (1q41 GAIN), IntClust 10 (8q24.21 GAIN, 5q11.2 LOSS), IntClust 5 (17q12 GAIN), IntClust 6 (8p12 LOSS), IntClust 7 (16q23.1 LOSS, 8q24.21 GAIN)

Supplementary Table 8. Distribution of BCCS subtypes of GSE76275 samples (n = 157) with respect to Burstein subtypes and TNBCtype4 subtypes

	NCS1 (n = 112)	NCS2 (n = 11)	NCS3 (n = 33)
Burstein subtypes[1]			
BLIA	44	0	6
BLIS	58	0	0
LAR	2	10	1
MES	8	1	26
TNBCtype4[2]			
BL1	45	0	1
BL2	19	1	11
LAR	3	10	6
M	35	0	1
UNK	10	0	14

[1] BLIA = Basal-Like Immune-Activated, BLIS = Basal-Like Immune-Suppressed, LAR = Luminal-AR, MES = Mesenchymal

[2] BL1 = basal-like 1, BL2 = basal-like 2, LAR = luminal AR, M = mesenchymal, UNK = unclassified

Supplementary Table 9. Distribution of GSE76275 samples (n = 157) in the TNBCtype4 subtypes and Burstein subtypes within the BCCS subtypes[1]

	TNBCtype4[2]				
Burstein Subtypes[3]	BL1	BL2	LAR	M	UNK
	BCCS NCS1 (n = 112)				
BLIA	26	13	0	0	5
BLIS	19	2	0	35	2
LAR	0	0	2	0	0
MES	0	4	1	0	3
	BCCS NCS2 (n = 11)				
BLIA	0	0	0	0	0
BLIS	0	0	0	0	0
LAR	0	0	10	0	0
MES	0	1	0	0	0
	BCCS NCS3 (n = 33)				
BLIA	1	1	0	0	4
BLIS	0	0	0	0	0
LAR	0	0	1	0	0
MES	0	10	5	1	10

[1] There was 1 BCCS tie

[2] BL1 = basal-like 1, BL2 = basal-like 2, LAR = luminal AR, M = mesenchymal, UNK = unclassified

[3] BLIA = Basal-Like Immune-Activated, BLIS = Basal-Like Immune-Suppressed, LAR = Luminal-AR, MES = Mesenchymal

Supplementary Table 10. Summary of the methods used to define the six subtype systems

Method	Gene Filtering	Cluster Method	Selection of Cluster Number
A	MAD > 0.5	hierarchical, average linkage	ConsensusClusterPlus 2-8 clusters, sample the genes
B	top 15% by var	hierarchical, ward linkage	ConsensusClusterPlus 2-8 clusters, sample the samples
C	varTest p < 0.01	hierarchical, ward linkage	ConsensusClusterPlus 2-8 clusters, sample samples and genes
D	top 5% by IQR	PAM, with varying k, Euclidean distance	gap statistic
E	sigma > 0.8, 0.5, 1, 1.1	NMF, run 30 times, 2-8 clusters	cophenetic correlation coefficient & heatmap
F	IQR > 1.2	same as method E	same as method E

Supplementary Table 11. Summary of the alternative methods to define six subtype systems

Method	Gene Filtering	Cluster Method	Selection of Cluster Number
I	standard deviation > 0.8	PAM, with varying k, Pearson distance	ConsensusClusterPlus 2-8 clusters, sample the samples
II	top 1,000 by MAD	NMF, k = 3, 50 iterations	cophenetic correlation coefficient & heatmap
III	top 5% by variance	fuzzy clustering, 2-5 clusters, pearson distance	average silhouette width
IV	varTest p < 0.01	hierarchical, average linkage	ConsensusClusterPlus 2-8 clusters, sample the genes
V	top 15% by var	hierarchical, ward linkage	ConsensusClusterPlus 2-8 clusters, sample the samples
VI	MAD > 0.5	hierarchical, ward linkage	ConsensusClusterPlus 2-8 clusters, sample samples and genes
